# Spatio-temporal trends of the age-at-menarche percentiles among Portuguese women since 1920

**DOI:** 10.1186/s12905-023-02608-5

**Published:** 2023-09-07

**Authors:** Vitor Rodrigues, Rui Martins, Bruno de Sousa

**Affiliations:** 1 Faculty of Medicine, University of Coimbra, Rua Larga, 3004-504 Coimbra, Portugal; 2 Liga Portuguesa Contra o Cancro, Núcleo Regional do Centro, Rua Dr. António José de Almeida, 329 - piso 2 - Sala 56, 3000-045 Coimbra, Portugal; 3 Departamento de Estatística e Investigação Operacional, Faculdade de Ciências, Universidade de Lisboa, Portugal; Centro de Estatística e Aplicações da Universidade de Lisboa (CEAUL), Lisboa, Portugal; 4 Faculty of Psychology and Education Sciences (FPCE); Center for Research in Neuropsychology and Cognitive and Behavioral Intervention (CINEICC), University of Coimbra, Coimbra, Portugal

**Keywords:** Menarche, GAMLSS, Distributional regression, Spatial, Temporal, Portugal

## Abstract

**Background:**

This work aims to study the spatio-temporal evolution of a woman’s age at menarche in the central region of Portugal. One of the concerns of the study is early or late menarches; thus, we consider percentile regression to build the respective curves as opposed to the more traditional mean regression approach.

**Methods:**

We analysed the data from $N=452\,348$ women born in the period 1920–1973 who attended a free breast cancer screening program between 1990 and 2019. Distributional regression models inside the package GAMLSS in R were considered. These methods allowed us not only to model the location (mean) of the specific probability distribution of the age at menarche, but also allowed for the scale (variance) parameter of this distribution to depend on covariates. Additionally, a spatial random-effect was considered in order to capture the correlation at the regional level. The obtained clustered spatial effects were analysed to assess geographical differences among the percentiles of the age at menarche by year of birth.

**Results:**

A decreasing trend in the age at menarche (about 1.5 years in 5 decades) and regional differences for all the considered percentiles were found. Women living in the north-central areas of the central region of Portugal tend to have menarche at older ages.

**Conclusion:**

We obtained percentile estimates for the age at menarche by year of birth and region of residence and demonstrated that these two explanatory variables have an impact on the explanation about the decreasing trend in age at a woman’s first menstruation.

## Content

Menarche, the term to denote the onset of menstruation, marks the beginning of a girl’s fertility. Later age at menarche (AaM) began to occur in the Modern Era, especially after the Industrial Revolution (1760–1840) possibly due to the deterioration of certain living conditions. Estimates for the second half of the 18th century point to an AaM between 15 and 16 years old [1]. This behaviour was found to undergo a reversal in the 20th century, with most studies showing a trend for earlier AaM in both developed and developing countries [2–8]. This feature has been associated with life-style, environment, socio-economic status, access to health care and higher levels of literacy [9]. Additionally, several studies point to an association with the consumption of energy-rich food (i.e. with an increased body mass index) [10, 11]. The work of Cabanes et al. 2009 [12], which studied women born from 1925 to 1962, along with Sohn et al. 2017 [13], studying women born in the period 1941 – 1992, noted some rates of decline in AaM. The European Prospective Investigation into Cancer & Nutrition (EPIC) study found that mean AaM decreased among female participants born from 1912 to 1964 in nine European countries [14]. However, in the past few decades, this trend is disappearing, with some studies showing a cessation of the decline [1]. In France, Lalys et al. [15] studied a group of school girls born between 1979 and 1994, and in Netherlands Talma et al. [16] studied girls born before the year 2000. Both studies concluded that there are signs of slowing down or stabilization. Finer and Philbin (2014) [17] report a mean AaM among US girls born in 1993 of 12.3 years of age – similar to those born in 1980.

The Portuguese panorama is identical to those already described above. A work [18] that analyses a Portuguese community of students at the University of Coimbra born in the period 1972–1983 claims that the mean AaM for girls born in 1983 was 12.03 years and that the place of residence during childhood and adolescence had a significant effect on the mean AaM. Girls from rural areas had a later menarche when compared to those who spent their childhood/adolescence in urban areas. Despite being focused on a specific-group, this work offers an advantage in our study, in analysing a time-period immediately following that considered in our study $(1920-1973)$, thus becoming an interesting point of comparison.

Age at menarche plays a very important role in the research about breast cancer, hence having important individual and public health implications. Early puberty has been associated with an increased risk of this type of cancer, but also with obesity and diabetes [19]. Burgess et al. [20] suggested a protective effect of later AaM on breast cancer risk.

It is well known that AaM varies between and within populations and is influenced by many factors. It may be delayed under poor socio-economic and health conditions or accelerated by residence in an urban community [21]. The starting point for the present research was the idea to understand the spatio-temporal evolution of menarche in the central region of Portugal. To achieve this goal we analysed the dataset on the Breast Cancer Screening Program provided by the Portuguese Cancer League (Liga Portuguesa Contra o Cancro – LPCC) in the Central region of Portugal [22].

A typical regression analysis, in most contexts, focuses on explaining the expected value dependency of a response variable as a function of a set of explanatory variables. However in some situations, if we want to understand not only the behaviour of the average person, but also the behaviour of those belonging to the extremes of the population, we might consider percentile regression [23, 24] which allows us to go beyond mean regression, enabling building regression curves for the percentiles, instead of the mean of a response variable. Rigby et al. [25] argue that for data with more than 1000 observations, regression models beyond the mean should be the norm, not the exception.

## Data description

The study of the AaM percentiles in the central region of Portugal was carried out considering the full dataset on the Breast Cancer Screening Program provided by the Portuguese Cancer League (LPCC) in the Central region of Portugal in the period $1990-2019$ which has screened $N=452\,348$ women born in the period $1920-1973$. At the age of 45 (since 2010 the age is 50) all women in each of the 89 municipalities (see Fig. 3) were invited to undergo a free screening mammogram and every two years thereafter until the age of 69. These regions roughly represent 25% of the Portuguese population. Although we must add a caveat here, because this spatial information is regarding only the current place of residence, and no other spatial information such as place of birth or where women grew up as a child is known. More details about the screening program and the inclusion criteria are given elsewhere [22, 26, 27].

**Fig. 1:**
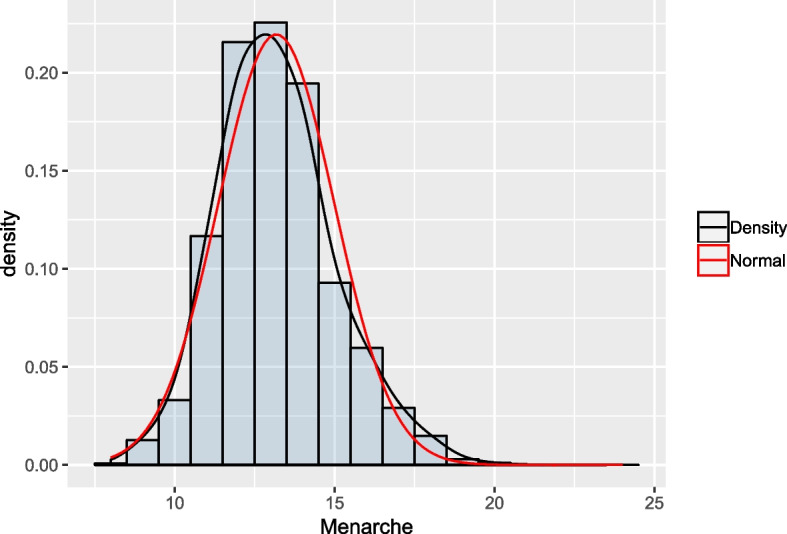
Age at menarche. The variable has a positive skewed distribution (black line), so one must consider a distribution allowing for positive skewness

Age at menarche (AaM) registered in years at the first interview, was the response variable of interest. The average was 13.18 with a minimum of 8 and a maximum of 24. We could raise the question about the existence of bias in the reported ages, but many studies have demonstrated that this reported age is quite accurate, most likely due to the emotional significance of menarche for a young girl [28]. Figure 1 shows the histogram for the unconditional distribution of this variable with a modal class at 13 years old. Summarized in Table 1 are its descriptive percentiles by decade from 1920 to 1973, and Table 2 depicts the mean and the median by municipality and decade. Year of birth (Byear) and the demographic information given by the municipality of residence (Muni) were the independent variables.

**Table 1:** Summary of the AaM percentiles by decade of birth since 1920 until 1973. Last column presents the total number of women screened per decade of birth

AaM (Percentiles)								
Decade	5	10	25	50	75	90	95	women
20’s	11	12	12	14	15	17	18	19844
30’s	11	11	12	14	15	17	17	72206
40’s	11	11	12	13	14	16	17	109335
50’s	11	11	12	13	14	15	16	122045
60’s	10	11	12	13	14	15	16	109266
70’s	10	11	12	12	14	15	15	19652

**Table 2:** Mean $(\bar{x})$ and median $(\tilde{x})$ for the AaM by municipality since 1920 until 1973 (Descriptive statistics)

	Decade							Decade					
	20’s	30’s	40’s	50’s	60’s	70’s		20’s	30’s	40’s	50’s	60’s	70’s
Municipality	$\bar{x}; \;\tilde{x}$	$\bar{x}; \;\tilde{x}$	$\bar{x}; \;\tilde{x}$	$\bar{x}; \;\tilde{x}$	$\bar{x}; \;\tilde{x}$	$\bar{x}; \;\tilde{x}$	Municipality	$\bar{x}; \;\tilde{x}$	$\bar{x}; \;\tilde{x}$	$\bar{x}; \;\tilde{x}$	$\bar{x}; \;\tilde{x}$	$\bar{x}; \;\tilde{x}$	$\bar{x}; \;\tilde{x}$
ÁGUEDA	14.12; 14	13.77; 14	13.48; 13	13.04; 13	12.79; 13	12.67; 12	MONTEMOR-O-VELHO	14.01; 14	13.71; 14	13.28; 13	12.85; 13	12.58; 13	12.63; 13
AGUIAR DA BEIRA	13.51; 13	13.59; 13	13.25; 13	12.79; 13	12.44; 12	12.54; 12	MORTÁGUA	14.71; 16	14.67; 14	14.18; 14	13.6; 13	13.1; 13	12.64; 13
ALB.-A-VELHA	14.61; 15	14.34; 14	13.88; 14	13.42; 13	13.02; 13	12.55; 13	MURTOSA	14.13; 14	13.73; 13	13.47; 13	13.21; 13	12.83; 13	12.49; 12
ALCOBAÇA	14.26; 14	14.02; 14	13.67; 14	13.21; 13	12.94; 13	–;–	NAZARÉ	14.01; 14	13.65; 13	13.49; 13	12.93; 13	12.74; 13	12.76; 13
ALMEIDA	14.28; 14	13.72; 14	13.57; 14	13.24; 13	12.71; 13	12.68; 13	NELAS	14.11; 14	13.5; 13	13.25; 13	12.74; 13	12.52; 12	12.41; 12
ALVAIÁZERE	13.95; 14	14.01; 14	13.5; 13	13.15; 13	12.8; 13	12.68; 13	OLEIROS	13.56; 13	13.49; 13	13.04; 13	12.76; 13	12.43; 12	12.39; 12
ANADIA	14.1; 13.5	14.07; 14	13.61; 13	13.18; 13	12.95; 13	12.82; 13	OLIVEIRA DE FRADES	13.85; 14	13.58; 14	13.18; 13	12.92; 13	12.6; 12.5	12.7; 13
ANSIÃO	–;–	13.93; 14	13.54; 13	13.14; 13	12.67; 13	12.04; 12	OLIV. DO BAIRRO	14.04; 14	13.79; 14	13.36; 13	12.89; 13	12.62; 12	12.6; 12.5
ARGANIL	13.92; 14	13.58; 13	13.17; 13	12.74; 13	12.51; 12	12.55; 12	OLIV. DO HOSPITAL	14.52; 14	14.16; 14	13.76; 14	13.32; 13	13.19; 13	–;–
AVEIRO	14.01; 14	13.72; 14	13.35; 13	12.95; 13	12.67; 13	–;–	OURÉM	13.83; 14	13.75; 14	13.33; 13	12.98; 13	12.67; 13	12.62; 12
BATALHA	–;–	13.64; 14	13.27; 13	12.93; 13	12.58; 12	12.8; 12.5	OVAR	12.69; 12	13.54; 13	13.21; 13	12.83; 13	12.66; 13	12.61; 12
BELMONTE	13.87; 14	14.1; 14	13.67; 13	13.22; 13	12.85; 13	12.65; 12	PAMPI. DA SERRA	14.58; 14	14.2; 14	13.57; 13	13.11; 13	12.85; 13	12.8; 13
CANTANHEDE	13.6; 13	13.36; 13	13; 13	12.77; 13	12.36; 12	12.49; 12	PEDROGÃO GRANDE	14.1; 14	13.96; 14	13.55; 14	13.08; 13	12.94; 13	12.81; 13
CARREGAL DO SAL	14.47; 14	14.26; 14	13.74; 14	13.32; 13	12.94; 13	12.7; 12.5	PENACOVA	13.56; 13	13.45; 13	13.34; 13	12.86; 13	12.61; 12	12.43; 12
CAST. DE PÊRA	14.33; 14	14.11; 14	13.71; 14	13.22; 13	12.66; 13	12.5; 12.5	PENALVA DO CASTELO	13.64; 14	13.54; 13	13.13; 13	12.79; 13	12.58; 12	–;–
CASTELO BRANCO	13.76; 14	13.7; 14	13.33; 13	13; 13	12.66; 13	12.7; 13	PENAMACOR	13.37; 13	13.41; 13	13; 13	12.63; 13	12.43; 12	12.36; 12
CASTRO DAIRE	14.34; 14	14.28; 14	13.74; 14	13.27; 13	12.88; 13	12.7; 13	PENEDONO	14.18; 14	14.08; 14	13.58; 13	13.15; 13	12.91; 13	12.81; 13
CELORICO DA BEIRA	14.26; 14	14.06; 14	13.62; 13	13.14; 13	12.8; 13	12.77; 13	PENELA	–;–	13.7; 14	13.41; 13	12.93; 13	12.94; 13	–;–
COIMBRA	–;–	13.84; 14	13.51; 13	12.9; 13	12.48; 12	12.65; 13	PINHEL	14.19; 14	13.87; 14	13.41; 13	12.92; 13	12.75; 13	12.75; 13
CONDEIXA-A-NOVA	13.88; 14	13.77; 14	13.37; 13	12.8; 13	12.54; 12	12.25; 12	POMBAL	14.62; 15	14.08; 14	13.76; 14	13.28; 13	12.96; 13	12.82; 13
COVILHÃ	–;–	13.79; 14	13.38; 13	12.89; 13	12.53; 12	13.3; 13	PORTO DE MÓS	14.27; 14	14.05; 14	13.54; 13	13.15; 13	12.96; 13	12.79; 13
ESTARREJA	14.01; 14	13.56; 13	13.23; 13	12.85; 13	12.7; 13	13.06; 13	PROENÇA-A-NOVA	13.88; 14	13.66; 13	13.27; 13	12.74; 13	12.5; 12	12.77; 12
FERREIRA DO ZÊZERE	14.27; 14	13.98; 14	13.45; 13	13; 13	12.83; 13	12.58; 12	RESENDE	14.35; 14	14.02; 14	13.61; 13	13.18; 13	12.62; 12	12.62; 13
FIGUEIRA DA FOZ	13.95; 14	13.57; 13	13.14; 13	12.71; 13	12.55; 12	12.62; 12	SABUGAL	13.94; 14	14; 14	13.42; 13	13.15; 13	12.63; 12	12.49; 12
FIG. CAST. RODRIGO	13.39; 13	13.61; 14	13.21; 13	12.83; 13	12.53; 12	12.27; 12	SANTA COMBA DÃO	14.4; 14	14.32; 14	13.87; 14	13.32; 13	12.82; 13	12.55; 12
FIGUEIRÓ DOS VINHOS	13.8; 14	13.88; 14	13.55; 13	13.3; 13	12.81; 13	12.66; 12	SÃO PEDRO DO SUL	14.27; 14	14.17; 14	13.97; 14	13.55; 13	12.92; 13	12.6; 12.5
FORNOS DE ALGODRES	14.54; 15	14.22; 14	13.57; 13	13.26; 13	12.95; 13	–;–	SÁTÃO	13.77; 14	13.45; 13	13.15; 13	12.71; 13	12.55; 12	12.65; 12
FUNDÃO	13.62; 13	13.35; 13	12.96; 13	12.7; 13	12.47; 12	12.43; 12	SEIA	14.38; 14	14.43; 14	13.99; 14	13.53; 13	13.14; 13	12.77; 13
GÓIS	13.93; 14	13.48; 13	13.24; 13	12.8; 13	12.48; 12	12.21; 12	SERNANCELHE	14.54; 14	14.51; 14	13.99; 14	13.48; 13	13.03; 13	13.24; 13
GOUVEIA	13.58; 14	13.64; 13	13.4; 13	13.01; 13	12.67; 13	12.63; 13	SERTÃ	13.8; 14	13.55; 13	13.16; 13	12.83; 13	12.55; 12	12.41; 12
GUARDA	14.22; 14	13.97; 14	13.61; 13	13.25; 13	12.82; 13	12.33; 12	SEVER DO VOUGA	14.6; 15	14.19; 14	13.74; 14	13.18; 13	12.84; 13	12.84; 13
IDANHA-A-NOVA	14.33; 14	14.2; 14	13.96; 14	13.34; 13	13.01; 13	12.89; 13	SOURE	13.81; 13	13.54; 13	13.32; 13	12.97; 13	12.63; 13	12.53; 12
ÍLHAVO	13.94; 14	13.88; 14	13.48; 13	12.89; 13	12.76; 13	12.49; 12	TÁBUA	13.31; 13	13.89; 14	13.36; 13	13.16; 13	12.72; 13	12.4; 12
LAMEGO	13.92; 14	13.73; 14	13.4; 13	13; 13	12.67; 13	12.48; 12	TOMAR	14.05; 14	13.97; 14	13.51; 13	13.18; 13	12.83; 13	12.91; 13
LEIRIA	13.61; 13	13.43; 13	13.02; 13	12.9; 13	12.46; 12	12.47; 13	TONDELA	13.59; 13	13.57; 13	13.11; 13	12.7; 13	12.46; 12	12.22; 12
LOUSÃ	13.73; 14	13.51; 13	13.14; 13	12.78; 13	12.42; 12	12.51; 12	TRANCOSO	14.19; 14	14; 14	13.55; 13	13.01; 13	12.72; 13	12.5; 12
MAÇÃO	14.44; 14	14.34; 14	14.06; 14	13.68; 13	–;–	–;–	VAGOS	13.4; 13	13.21; 13	12.89; 13	12.82; 13	12.54; 12	12.21; 12
MANGUALDE	14.73; 15	14.6; 14.5	13.83; 14	13.48; 13	12.99; 13	–;–	V. DE REI	14.29; 14	14.09; 14	13.54; 13	13.13; 13	12.73; 13	12.81; 13
MANTEIGAS	15; 14	13.77; 14	13.45; 13	13.12; 13	12.78; 13	12.93; 13	V. NOVA DE FOZ CÔA	13.76; 14	13.66; 13	13.29; 13	12.85; 13	12.63; 13	12.53; 12
MARINHA GRANDE	14.04; 14	14.01; 14	13.55; 13	13.03; 13	13.11; 13	–;–	V. NOVA DE PAIVA	13.98; 14	13.23; 13	13.03; 13	12.59; 12	12.53; 12	11.8; 11
MEALHADA	14.03; 14	13.79; 14	13.37; 13	12.93; 13	12.6; 13	12.39; 12	V. NOVA DE POIARES	13.86; 14	13.72; 14	13.35; 13	12.86; 13	12.53; 12	12.69; 13
MÊDA	13.88; 14	13.59; 14	13.25; 13	12.81; 13	12.54; 13	–;–	V. VELHA DE RODÃO	14.04; 14	13.95; 14	13.53; 13	13.15; 13	13.04; 13	–;–
MIRA	14.12; 14	13.48; 13	13.2; 13	12.84; 13	12.6; 12	12.6; 13	VISEU	13.97; 14	13.88; 14	13.48; 13	13.06; 13	12.68; 13	12.64; 13
MIRANDA DO CORVO	13.39; 13	13.1; 13	13.02; 13	12.64; 13	12.5; 12	12.5; 12	VOUZELA	14.48; 14	14.46; 14	13.82; 14	13.38; 13	13.01; 13	12.54; 13
MOIMENTA DA BEIRA	14.29; 14	14.02; 14	13.64; 14	13.11; 13	12.84; 13	12.72; 13							

## Methods

A typical regression analysis has the advantage of being very well known to the users, although it is very likely that in many scenarios other properties of the response distribution (e.g. the variance) may also depend on covariates. Besides this, one may want to comprehend not only the behaviour of the average person, but the behaviour of those belonging to the population’s extremes. To account for these features, we will consider a statistical framework developed within the context of Generalized Additive Models for Location, Scale and Shape (GAMLSS) [29], also known as distributional regression models [30]. These models have several advantages, but for our study the principal characteristic is that it assumes a known parametric family of distributions for the response variable which allows an easy calculation of the conditional percentile curves of the AaM given birth year and municipality of residence. Furthermore, the covariate effects of interest can be of flexible forms (e.g. smoothing functions) and not restricted to the traditional, and perhaps unrealistic, linear effect. At the same time, it ensures that the adjusted percentile curves do not cross. A competing method is quantile regression [31], where the adjusted curves might cross, because it does not assume a distribution for the response variable and, therefore, can be considered in the realm of non-parametric methods [25]. For their part, other authors have been considering other approaches. Most use simple statistical methods like ANOVA [32] or linear regression [12]. Chumlea et al. [33] considered a probit analysis.

### Statistical models

Based on data for $i = 1, \ldots , N=452\,348$ women, we will assume conditional independence of the individual ages at menarche, AaM, given the covariates Byear and Muni.

An initial exploratory analysis showed that the distribution of the AaM is right skewed (*vide* Fig. 1), and a non-linear assumption for the relation between the AaM and Byear is better supported by the data. Taking this into consideration, we opted to analyse the data with a model within the aforementioned class – GAMLSS. This method offers a highly flexible approach in that constraints to the traditional distributional assumptions, such as normality, are removed. The method enables the use of skewed distributions without having to transform the data, allowing us to work on the scale of the data, which is a very important feature. Additionally, as already said, all the parameters of the response probability distribution can be modelled by explanatory variables and not only the location. For instance, it is possible to model the variance and the skewness (for some distributions it is also possible to model the kurtosis). All the explanatory variables are introduced into the model parameters through predictors, which can be linear functions of the explanatory variables or can take the form of structured additive predictors with non-linear or smoothing functions of explanatory variables.

The generalized Akaike Information Criterion (GAIC), a model selection measure, was considered to determine the best fitting distribution of the data. It was found that the *Box-Cox Cole and Green* distribution, $\text {BCCG}(\mu ,\sigma ,\nu )$, provides the lower GAIC value (i.e. the best fit) when compared to other alternative distributions with continuous support. Specifically, we also considered the Normal, the t and the Box-Cox Power Exponential distributions, which are all available in GAMLS. The main statistical model for analysing the data within the GAMLSS framework that we will be dealing with is:

1 $$\begin{aligned} \texttt {AaM}\sim & {} \text {BCCG}(\mu ,\sigma ,\nu ),\nonumber \\ \mu= & {} \beta _{10} + f_{11}(\texttt {Byear}) + f_{12}(\texttt {Muni}), \nonumber \\ \log {(\sigma )}= & {} \beta _{20} + f_{21}(\texttt {Byear}) + f_{22}(\texttt {Muni}), \nonumber \\ \nu= & {} \beta _{30}. \end{aligned}$$

We defined an additive model for the location parameter, $\mu$, which for this distribution represents its median, a very relevant parameter in our work, as we are interested in estimating the percentiles. The linear predictor defined as $\mu = \beta _{10} + f_{11}(\texttt {Byear}) + f_{12}(\texttt {Muni})$ allows for directly modelling the median. For the scale of the response variable, $\sigma$, we let the parameter depend on the explanatory variables Byear and Muni. It is a multiplicative model resulting from the log-link, which in turn ensures positive values for the parameter. For this distribution the scale parameter is approximately the coefficient of variation. The skewness parameter, $\nu$, is modelled only with an intercept term. It was found that adding covariates (e.g. age) to its linear predictor did not improve the adjustment because those models produced greater GAIC values.

The functions $f_{11}$ and $f_{21}$, modelled as cubic splines, represent the temporal effects of the year of birth, and $f_{12}$ and $f_{22}$ are the spatially correlated effects of the residence municipalities modelled as an intrinsic autoregressive process (IAR), a limiting case of the conditional autoregressive models (CAR) of Besag et al. [34]. Thus, we are considering that the spatial effect is a Markov random field (MRF), i.e. assuming that the spatial random effects (our spatial variables) have a joint distribution which is specified by considering conditional independence locally. The IAR models are a typical choice when dealing with a dependent variable observed in geographical areas sharing borders, because we expect neighbouring areas to have more similar observed values for the AaM than areas farther apart. The consequence of this approach is that the parameter estimates for neighbouring locations are shrinked towards its mean.

## Results

The conditional percentile values were thus obtained via the R software (v 4.1.0), namely using the main package gamlss (version 5.1-4) and two additional packages that provide a set of functions to fit models with spatial variables (gamlss.spatial and gamlss.add).

The chosen model is written below in terms of the R syntax based on the gamlss package:
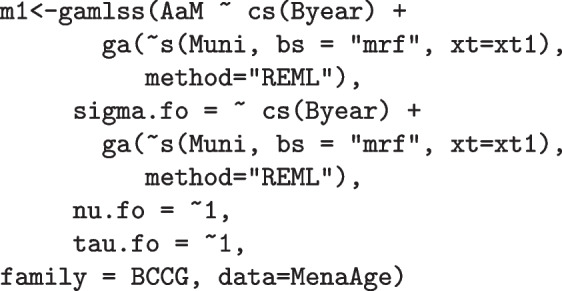


### Age at menarche – temporal trends

**Fig. 2:**
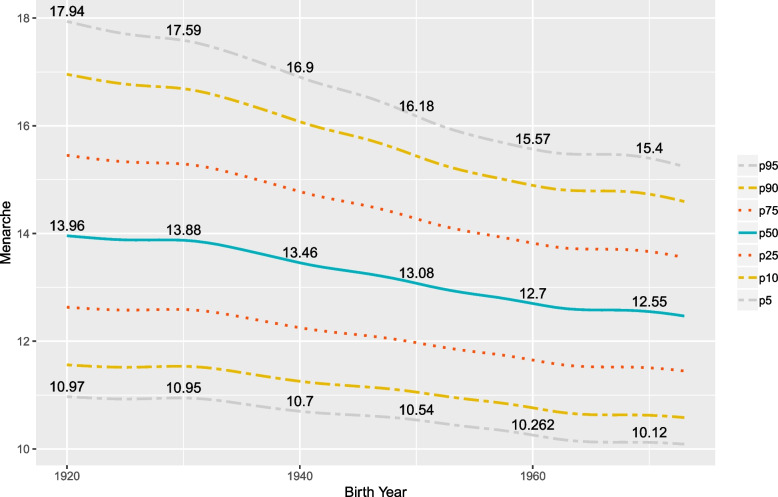
Estimated percentile curves for the AaM by year of birth since 1920. The values printed are for the percentiles 5%, 50% and 95% for the years $1920,1930,\ldots ,1970$

A first distributional regression model without covariates was considered in order to estimate the median AaM for the overall population since 1920, resulting in a point estimate of 13.05 years old. Then we limited the model to one explanatory variable Byear for exploring the temporal trends in the central region of Portugal. It should be noted that the percentiles curves displayed in Fig. 2 are not linear. This behaviour could not be captured within a typical linear regression analysis and is facilitated by the non-linear approach permitted by the generalized additive models. Additionally, the percentiles 25%, 50%, 75%, 90% and 95% are steadily decreasing at a greater rate since 1920 than the percentiles 5% and 10%, which could mean that there is not much more space for reductions in the menarche age for the girls with the earliest menarches. The stagnation trend reported by other studies also appears to be emerging, mainly in the 1960s. From 1970 onwards the downward trend reappears, which is in line with the results published in [18].

### Age at menarche – spatio-temporal trends

**Fig. 3:**
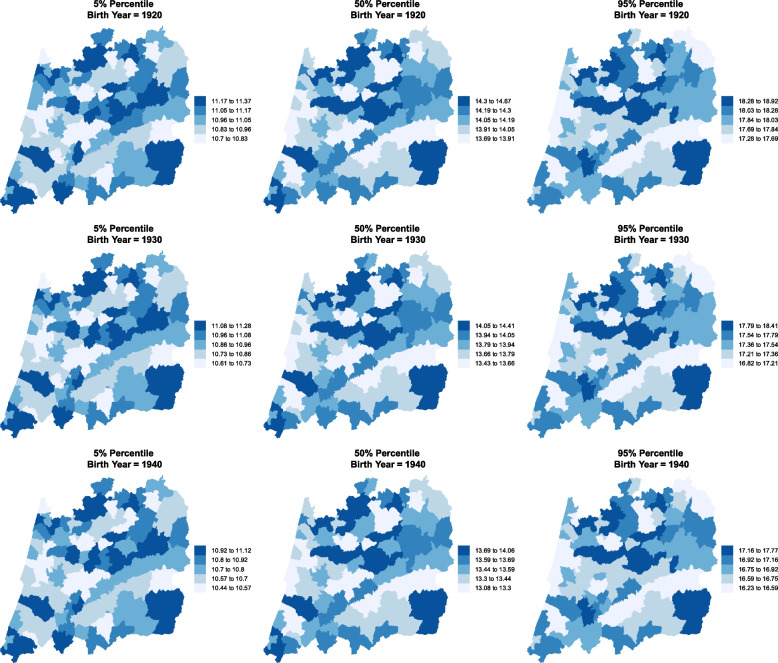
Estimated AaM by region and birth year. Percentiles 5%, 50% and 95% for the central region of Portugal for the years 1920, 1930 and 1940

**Fig. 4:**
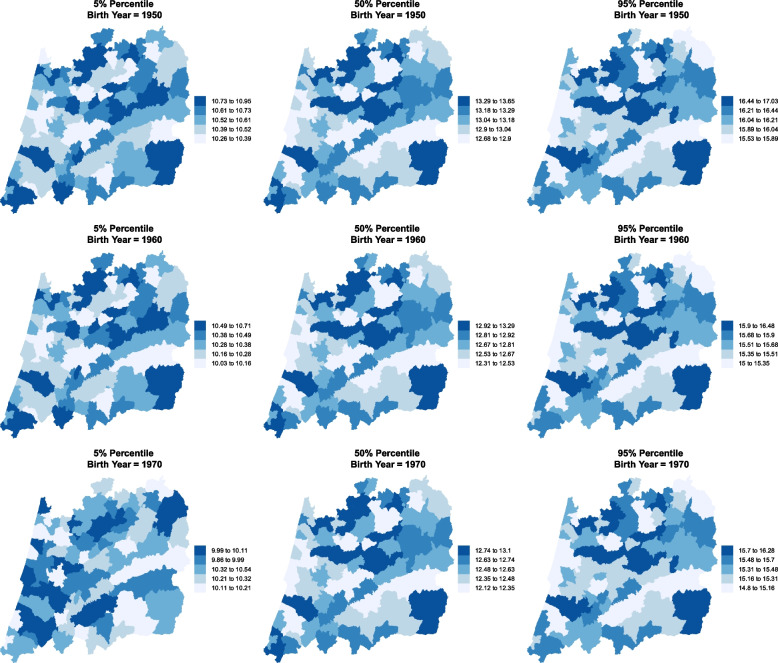
Estimated AaM by region and birth year. Percentiles 5%, 50% and 95% for the central region of Portugal for the years 1950, 1960 and 1970

Each municipality is likely to have the median and the variability of the AaM close to those of its neighbours. Bearing this in mind, spatial effect, $f_{12}(\texttt {Muni})$ and $f_{22}(\texttt {Muni})$, were added to the model in (1) aiming at capturing the influence of neighbouring locations not available by the observed covariates. Figures 3 and 4 present the spatio-temporal trends in the percentiles for the AaM, which are already accounting for the spatial dependency of the response variable (AaM). It is clear that the patterns through the decades have remained unchanged, i.e. regions with the larger values in 1920 maintain their position in 1970, although all have being decreasing, with only a few exceptions. The northern municipalities tend to present larger values for all the considered percentiles.

**Fig. 5:**
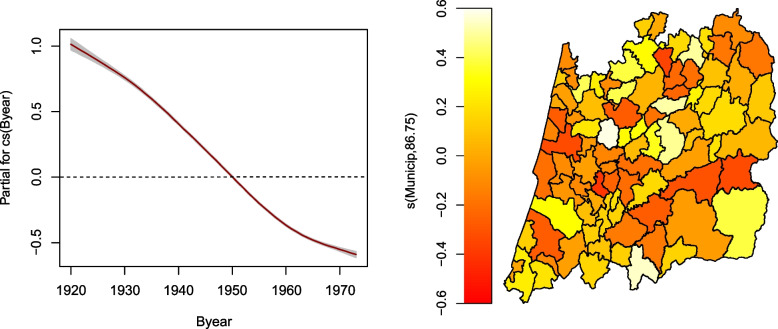
Estimated effects for the AaM model showing the temporal variation of the birth year effects (left) together with the clustered spatial effects (right)

Figure 5 depicts the effects of each of the covariates (Byear and Muni) on the AaM location parameter, i.e. its median. The interpretation is relatively straightforward. A positive time effect was found until approximately 1950 and thereafter a negative effect. This means that a woman born before 1950 had an AaM above the median for the overall population and accounting all the years, i.e. 13.05, and a woman born after 1950 will tend to have an AaM below 13.05. For the spatial effects, a municipality with a negative effect means that women residing there have ages at menarche below the median when comparing to the overall population. On the other side, areas with a positive spatial effect will tend to have ages at menarche above the median of the overall population. Looking to Figs. 3 and 4 and comparing them to Fig. 5, it is clear that larger values of the median AaM are associated with a positive spatial effect and *vice-versa*.

Another interesting feature of the temporal effect for the year of birth is obtained considering the getPEF function (Partial Effect function) which allows us to calculate the slope of the curve at each year (*vide* Table 3). For example, for the year 1960, in gamlss syntax this can be done by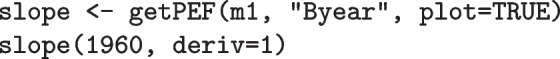 and the result is the slope of the curve’s tangent for the year 1960, which is not very different from the other years, at least until 1960. The result is approximately $-0.04$, meaning that for that year the median AaM was reduced at a rate of $0.04\times 365\approx 14.6$ days, or 4.87 months per decade. This value is in accordance with the findings of other studies, e.g. [35] which examines the evolution of the menarche in the period 1830–1960 in Europe and reported a decreasing of about 3 months per decade. For the year 1970, we have seen from Fig. 2 a curve with a horizontal aspect, meaning a stabilization. Calculating the slope at this time, the value of $-0.013$ appears, meaning that the median AaM is reducing in this year at a rate of $0.013\times 365=4.745$ days, or 1.58 months per decade, pointing toa slowing down in the reduction rate.

**Table 3:** Estimated decreasing rates per year (in days) of the median AaM for girls born since 1920 up to 1970

Birth Year	Decreasing Rate
1920	8.91
1925	9.1
1930	10.47
1935	13.04
1940	14.43
1945	14.71
1950	15.23
1955	13.72
1960	10.48
1965	6.26
1970	4.75

## Discussion

Estimation of the percentile curves for a response variable is widely used in medicine for checking whether an individual has an abnormally low or high value of the response variable (given the covariates of interest), and hence whether she/he is potentially at risk. For example, let us think of a region where breast cancer risk is high and that girls living there tend to have an early menarche when compared to the rest of the country. And suppose that all other characteristics generally known for being linked to breast cancer are equal across that country. That specific region may be the target of differentiated public policies.

The dataset here analysed is based on the largest sample size reported to date in Portugal, although it does not include women born in the last three decades, meaning that the percentiles of these youngest women cannot be ascertained. Nevertheless, and to the best of our knowledge, this study is the first to analyse and produce percentile curves for the women residing in the Central Portugal since 1920 and to use a distributional regression approach.

Probably not all spatial effects are being correctly captured because the spatial information in the data set regards the place of residence at the time of the participation in the screening program, i.e. when the women are adults, as already mentioned in the data description. However, we assume that these women have always been living there. This is more likely to be true for the interior areas than for the areas closest to the coast given that municipalities in Central and Eastern Portugal have lower levels of wealth. Despite the above, we do believe that the spatial results are quite robust because of the dimension of the sample here analysed. That said, some works, e.g. [36] show that the place of residence, and even the altitude of the residence area [37] during childhood, are likely to be more effective in estimating regional differences for the AaM.

The probability distribution that we chose for describing the AaM, $\text {BCCG}(\mu ,\sigma ,\nu )$, has several advantages for have being considered here. First its location parameter, $\mu$, is interpreted as the median of the distribution, which when describing the percentiles is a benefit. Additionally, we are free to control the shape parameter, $\nu$, representing the amount of skewness of the distribution, and from Fig. 1 we know that our distribution is positive skewed.

## Conclusion

We resorted to a distributional regression model to estimate several spatio-temporal percentiles for the AaM in the central region of Portugal considering a long period of time and using a representative sample with a large number of observations. The results show that within the period $1920-1973$, all the percentiles obtained for the AaM depict a decreasing trend. For a girl born in 1920, we would expect a median AaM of around 14 years. For a girl born in 1973, this value is about 1.5 years lower (Fig. 2). Interestingly, a pattern of AaM distribution by municipality emerges. Although all its percentiles are decreasing, the regions with the highest percentile values for AaM in 1920 are the same for 1970 (Figs. 3 and 4), with only a few exceptions.

In the middle of the 20th century the decreasing rate achieved its maximum of around 15.23 days per year (Table 3). This trend after 1973 seems to be slowing down or even stopping. We should note that the median’s rate of decrease is not replicated by the other percentiles. For instance, percentiles 90% and 95% decreased about 2.6 years in the span of 50 years. On the other hand, percentiles 5% and 10% decreased only about 0.8 years in the same range of 50 years. Early menarches (below percentile 5%) occur in the coastal (western) area of Portugal’s central region for all the decades considered and later menarches (above percentile 95%) occur in the central-north area.

## Data Availability

The data sets used and/or analysed during the current study are available from the corresponding author on reasonable request.
